# Genome-wide DNA methylation-analysis of blastic plasmacytoid dendritic cell neoplasm identifies distinct molecular features

**DOI:** 10.1038/s41375-024-02240-8

**Published:** 2024-04-10

**Authors:** Axel Künstner, Julian Schwarting, Hanno M. Witte, Pengwei Xing, Veronica Bernard, Stephanie Stölting, Philipp Lohneis, Florian Janke, Maede Salehi, Xingqi Chen, Kathrin Kusch, Holger Sültmann, Emil Chteinberg, Anja Fischer, Reiner Siebert, Nikolas von Bubnoff, Hartmut Merz, Hauke Busch, Alfred C. Feller, Niklas Gebauer

**Affiliations:** 1 Medical Systems Biology Group, University of Lübeck, Ratzeburger Allee 160, 23538 Lübeck, Germany; 2 University Cancer Center Schleswig-Holstein, University Hospital of Schleswig-Holstein, Campus Lübeck, 23538 Lübeck, Germany; 3 Department of Hematology and Oncology, University Hospital of Schleswig-Holstein, Campus Lübeck, Ratzeburger Allee 160, 23538 Lübeck, Germany; 4 Hämatopathologie Lübeck, Consultation Centre for Lymph Node Pathology and Hematopathology, 23562 Lübeck, Germany; 5 Department of Hematology and Oncology, Federal Armed Forces Hospital Ulm, Oberer Eselsberg 40, 89081 Ulm, Germany; 6 Department of Immunology, Genetics and Pathology, Uppsala University, 751 85 Uppsala, Sweden; 7 Division of Cancer Genome Research, German Cancer Research Center (DKFZ), 69120 Heidelberg, Germany; 8 German Cancer Consortium (DKTK), 69120 Heidelberg, Germany; 9 Institute of Human Genetics Ulm University and Ulm University Medical Center, 89081 Ulm, Germany

**Keywords:** Acute myeloid leukaemia, Cancer epigenetics

## Abstract

Blastic plasmacytoid dendritic cell neoplasm (BPDCN) constitutes a rare and aggressive malignancy originating from plasmacytoid dendritic cells (pDCs) with a primarily cutaneous tropism followed by dissemination to the bone marrow and other organs. We conducted a genome-wide analysis of the tumor methylome in an extended cohort of 45 BPDCN patients supplemented by WES and RNA-seq as well as ATAC-seq on selected cases. We determined the BPDCN DNA methylation profile and observed a dramatic loss of DNA methylation during malignant transformation from early and mature DCs towards BPDCN. DNA methylation profiles further differentiate between BPDCN, AML, CMML, and T-ALL exhibiting the most striking global demethylation, mitotic stress, and merely localized DNA hypermethylation in BPDCN resulting in pronounced inactivation of tumor suppressor genes by comparison. DNA methylation-based analysis of the tumor microenvironment by MethylCIBERSORT yielded two, prognostically relevant clusters (IC1 and IC2) with specific cellular composition and mutational spectra. Further, the transcriptional subgroups of BPDCN (C1 and C2) differ by DNA methylation signatures in interleukin/inflammatory signaling genes but also by higher transcription factor activity of JAK-STAT and NFkB signaling in C2 in contrast to an EZH2 dependence in C1-BPDCN. Our integrative characterization of BPDCN offers novel molecular insights and potential diagnostic applications.

## Introduction

Blastic plasmacytoid dendritic cell neoplasm (BPDCN) is an aggressive and extremely rare blood cancer, accounting for ~0.5% of acute hematological malignancies. In its recent editions, the WHO classification of myeloid neoplasms recognizes BPDCN as a distinct entity descending from non-activated, CD56^+^ plasmacytoid dendritic cells (pDC) [1–3]. However, a broader cellular origin encompassing transcriptional signatures of both AXL1^+^ SIGLEC6^+^ DCs and earlier, common dendritic cells, termed transitional DCs and conventional DCs, respectively, has been proposed, before, suggesting a diverse cellular ontogeny [4–6]. Clinically, skin lesions commonly precede bone marrow infiltration and secondary propagation into lymph nodes and extranodal organs. A striking 4:1 male predominance, attributed to sex-biased *ZRSR2* mutations and enrichment in elderly patients with a median age of around 70 years at diagnosis has been observed [7, 8]. While the typical BPDCN immunophenotype (CD4^+^, CD56^+^, CD123^+^) is relatively specific and reliably enables correct diagnosis, discrimination from AML, especially in cases with pDC features can be challenging [9, 10]. Investigation of pDC–associated antigens (e.g., TCL1 or CD303) can facilitate differential diagnosis, while the expression of B- (CD79b), T- (CD2, CD7), and precursor antigens (Tdt) poses variable pitfalls in both immunohistochemistry and flow cytometry [11, 12]. Treatment with conventional chemotherapy alone results in insufficient and short-lived remissions (median overall survival (OS) of 12 to 14 months), which necessitates either allogeneic or autologous stem cell transplantation in therapeutic approaches of curative intent [7, 13, 14]. Only through the introduction of tagraxofusp, a CD123-directed cytotoxin, which recently demonstrated high clinical efficacy, curative treatment has become possible in elderly patients, as well, while the outcome in relapsed/refractory cases remains dismal [15].

Genome-wide DNA methylation profiling has evolved from a descriptive, ontological analysis into a diagnostic assay of prognostic relevance across a variety of solid cancers, including CNS tumors and sarcomas [16, 17]. While genomic and transcriptional profiling has revolutionized the taxonomy of myeloid cancers, embedded in the current WHO classification, the study of DNA methylation may add another layer of insight into BPDCN biology that is so far insufficiently captured by DNA- and RNA-sequencing [18]. Methylome analysis may further assist in differential diagnostics between BPDCN and other malignancies sharing close molecular ties including chronic myelomonocytic leukemia (CMML), acute myeloid leukemia (AML), and myelodysplastic syndromes (MDS), which may occur syn- and metachronously in up to 20% of cases [19–21].

DNA methylation profiling can further assess the tumor immune microenvironment (TME), by deconvoluting cell types from within tissue-derived bulk DNA and discriminating immunologically hot from cold tumors [22]. The robust correlation of its output with an immunohistochemical dissection of the TME has been shown in non-small cell lung cancer and others [22, 23].

In this study, we have extended our previously published BPDCN cohort [6], assessed by paired whole-exome (WES) and transcriptome sequencing (RNA-seq) as well as genome-wide copy number analysis and conducted array-based genome-wide DNA methylation profiling and ATAC-sequencing on selected cases, allowing for a more profound and novel understanding of BPDCN pathobiology and reliable discrimination from AML as the predominant diagnostic challenge in clinical practice. Moreover, we identify two immunological subtypes, characterized by features in the TME and recurrent genomic alterations.

## Materials and methods

### Case selection, clinicopathological assessment, whole exome, and whole transcriptome sequencing processing and downstream including statistical analysis

For details on the above methods including transcription factor (TF) and pathway activities from RNA-seq data please see *Supplementary materials and methods*.

### Genome-wide DNA methylation profiling and data analysis

Whole-genome DNA methylation analysis was carried out on all 54 cases of the study cohort employing the Illumina EPIC array at ATLAS Biolabs. Bioconductor R package minifi (v1.46.0) was used to further process raw IDATs that were previously generated from iScan. The quality of samples was checked by using mean detection *P* values and only samples with *P* values < 0.05 were kept for further processing (five samples excluded). In addition, according to in-house bioinformatic QC pipelines were applied (one additional case was excluded). The remaining samples were normalized using quantile normalization (function *preprocessQuantile*) and DNA methylation data predicted sex was compared to the actual sex. Samples, where the predicted sex did not match with the actual sex, were removed (four samples removed), leaving 45 samples for further analysis. DNA methylation probes were quality filtered and probes with non-significant *P* values were removed (*P* > 0.01). Additionally, cross-reactive probes and BOWTIE2 multi-mapped probes were removed, and M- and beta-values of the remaining probes were extracted [24].

Differentially methylated probes between two conditions were identified using a linear modeling approach as implemented in limma. Generalized gene set testing (GST) on differentially methylated probes was performed by applying the *gsameth* function (missMethyl package v1.34.0) against the REACTOME and/or HALLMARK gene sets (MSigDB v7.5).

Details on the comparative analysis of genome-wide DNA methylation data from BPDCN (including DNA-methylation-based mitotic clock estimation), different sorted cell types, and data from AML and other entities including CMML, T-ALL, and malignant melanoma as well as the analysis of the tumor microenvironment (TME) by MethylCIBERSORT and immunohistochemistry are provided within the *Supplementary materials and methods*.

### FFPE-ATAC-seq

ATAC-sequencing on FFPE tissue sections from four typical pDC-like BPDCN patients was performed as described [25]. Briefly, for nuclei isolation 20 µm-thick sections were deparaffined and underwent subsequent enzyme digestion. Then, 50,000 isolated FFPE nuclei were used in each FFPE-ATAC reaction composed of Tn5-mediated transposition and T7 in vitro transcription. FFPE-ATAC libraries were then sequenced on an Illumina NovaSeq 6000 platform at Novogene (Cambridge, UK) to a depth of at least 40 million 150 bp single-end or paired-end sequencing reads per library.

## Results

### Clinical characteristics of the study group and expanded deconvolution cohort

Baseline clinicopathological characteristics of BPDCN cases included in the current study are briefly summarized in Table 1. Clinical outcomes, when available, reflected previous dismal observations in BPDCN with a median progression-free and OS of 8 and 12 months, respectively. Following our previous study, RNA-seq data were deconvoluted according to single-cell DC and monocyte datasets (Supplementary Fig. 1), and previous observations were recapitulated/extended to new samples including differentially mutated genes (Supplementary Fig. 2). Additionally, mutational landscape, MutSigCV analysis, and immunohistochemical profiles were extended to new cases as described (Supplementary Tables 1, 2, and 3) [6]. Tumor mutational burden (TMB) was confirmed to be significantly higher (*p* = 0.0055) among C1-BPDCN and the set of differentially mutated genes was updated (Supplementary Fig. 3a, b).

**Table 1:** Baseline clinicopathological characteristics of the study group.

Characteristics	BPDCN-C1 (*n* = 29)	BPDCN-C2 (*n* = 25)
Age (yrs.; median (range))	70 (15–91)	74 (42–90)
Sex		
Female	6 (21%)	8 (32%)
Male	23 (79%)	17 (68%)
Manifestation		
Skin (histologically confirmed)	20 (69%)	14 (56%)
Skin (suspected not biopsied)	6 (21%)	7 (28%)
Bone marrow	8 (28%)	8 (32%)
0 EN-sites	3 (10%)	3 (12%)
1–2 EN sites	26 (90%)	19 (76%)
>2 EN sites	–	3 (12%)
Stage (Ann Arbor)		
I/II	2/16 (13%)	3/16 (19%)
III/IV	14/16 (87%)	13/16 (81%)
ECOG PS		
0–1	8/10 (80%)	3/11 (27%)
≥2	2/10 (20%)	8/11 (73%)
B-symptoms		
No	6/14 (43%)	4/15 (27%)
Yes	8/14 (57%)	11/15 (73%)
Immunohistochemistry		
BPDCN-specific	29/29 (100%)	25/25 (100%)
CD56^+^	29/29 (100%)	25/25 (100%)
CD123^+^	29/29 (100%)	25/25 (100%)
TCL1	27/28 (96%)	17/23 (74%)
Immature lineage marker	21/29 (72%)	19/24 (79%)
CD34	1^a^/29 (3%)	4^a^/24 (17%)
TdT^+^	21/29 (72%)	16/23 (70%)
T-lineage markers	28/29 (96%)	24/25 (96%)
CD2^+^	5/22 (23%)	7/16 (44%)
CD3^+^	4/26 (15%)	5/23 (22%)
CD4^+^	28/29 (96%)	23/25 (92%)
B-lineage marker CD79A^+^	23/28 (82%)	20/23 (87%)
Myeloid-lineage markers	24/26 (92%)	21/23 (91%)
CD33^+^	24/26 (92%)	20/23 (87%)
CD117^+^	2/26 (8%)	9/23 (39%)
MPO^+^	1^a^/28 (4%)	4^a^/23 (17%)
Ki-67 (median, range)	60% (30–90%)	50% (25–90%)

*BPDCN* blastic plasmacytoid dendritic cell neoplasm, *ECOG* Eastern Cooperative Oncology Group, *EN* extranodal, *MPO* myeloperoxidase, *yrs* years.

^a^Cases with concurrent other myeloid neoplasia (CMML or AML; *n* = 4) or minimal positivity in a subclonal population (*n* = 1).

### Epigenetic profiling reveals significant deregulation of key regulatory pathways through loss of DNA methylation compared to dendritic cells

**Fig. 1:**
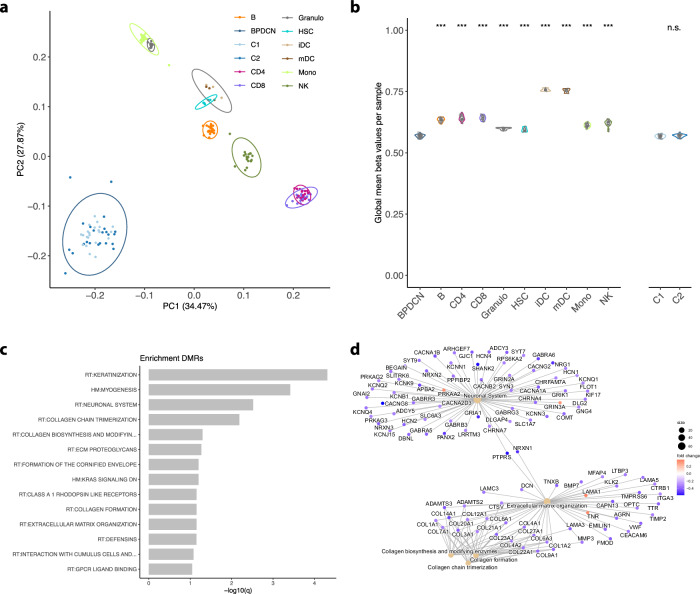
Epigenetic profiles of BPDCN and sorted hematopoietic cell populations. **a** First and second principal components of the 10,000 most variable DNA methylation sites in BPDCN (C1 and C2) and various hematopoietic cell types (*B* B lymphocytes, Granulo granulocytes, HSC hematopoietic stem cells, iDC immature dendritic cells, mDC mature dendritic cells, Mono monocytes, NK natural killer cells; ellipses show 95% confidence intervals of multivariate normal distribution). **b** Average genome-wide DNA methylation level (beta values) of BPDCN and various cell types and of BPDCN cluster C1 and C2. Individual estimates are shown as dots and cell type-specific distributions are shown as box- and violin-plots; significant differences against BPDCN were assessed by unpaired Wilcoxon test and significant levels are indicated by asterisks (*p < 0.05, **p < 0.01, and ***p < 0.001). **c** Enrichment analysis of differentially methylated regions (DMRs) between BPDCN and dendritic cells (harmonic mean of the individual CpG FDRs <0.01, absolute difference above 0.3) against HALLMARK and REACTOME gene sets. Only significant gene sets are shown (FDR < 0.1). **d** Network enrichment against REACTOME for DMRs (as in **c**); fold changes of DMRs (BPDCN vs DC) are color-scaled (red: higher DNA methylation in BPDCN; blue: higher DNA methylation in DC) and gene sets are denoted by light-brown nodes.

To assess epigenetic processes contributing to the malignant transformation from DCs to BPDCN and to allocate the entity within the spectrum of blood cells, we performed a principal component analysis (PCA) of BPDCN and various cell types of the peripheral blood (Fig. 1) [26]. Expectedly, we observed a clear segregation of blood cell types and BPDCN, but beyond this, a marked difference between BPDCN and previously profiled DC subsets became apparent (Fig. 1a) [27]. This was further reflected in significantly higher global mean DNA methylation levels in both early and mature DCs, signifying the extent of DNA methylation loss during transformation of DCs towards BPDCN (Fig. 1b). Enrichment analysis of differentially methylated regions (DMRs)(CpG FDRs <0.01, absolute difference above 0.3, enrichment analysis against HALLMARK and REACTOME) revealed significantly reduced DNA methylation in genes involved in extracellular matrix organization, collagen modulation, and neuronal systems. Oncogenic driver processes affected by these altered profiles included KRAS signaling (Fig. 1c, d).

### BPDCN is characterized by a DNA methylation profile distinct from its related entities yet borderline cases exist

**Fig. 2:**
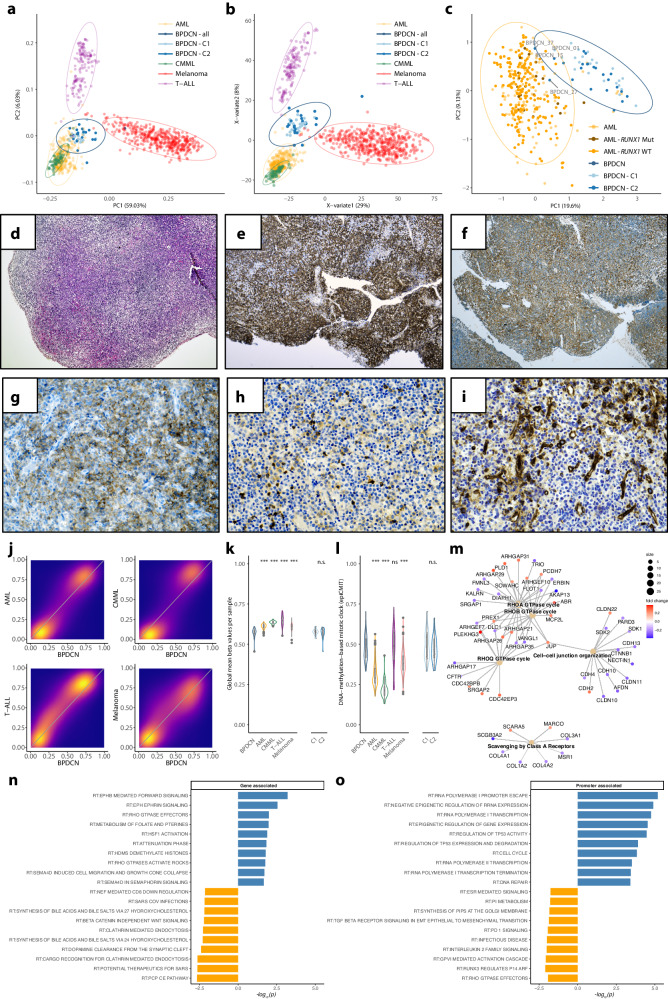
BPDCN DNA methylation in comparison to AML, CMML, t-ALL, and melanoma. **a** Visualization of the first and second principal components of the 10,000 most variable DNA methylation sites (ellipses show 95% confidence intervals of multivariate normal distribution). **b** Partial-least squares discriminant analysis (PLS-DA) of adjusted beta values. **c** First and second principal components of the comparison between BPDCN and AML (*RUNX1* wild-type and mutated samples highlighted differently). The four BPDCN cases falling inside the 95% confidence interval of the AML data are labeled. **d**–**j** a prototypical borderline case with both typical BPDCN as well as AML with pDC-like features. **d** Morphology of the neoplastic infiltrate within the lymph node resembles acute leukemia with polymorphic blast-like cells of variable size (H&E, 40×). **e** Uniform expression of CD123 initially led to the inclusion of BPDCN into the differential diagnosis (CD123, 40×). **f** Further immunophenotypic work-up revealed several atypical features, reminiscent of AML with partial pDC phenotype, including variable expression of CD33 in a significant fraction of the malignant infiltrate (CD33, 40×), yet only partial expression of CD56 (**g**; CD56, 200×) and CD117 (**h**; CD117, 200×). **i** The bi-phenotypic character of the infiltrate is further underlined by a strong CD34 expression of a minor fraction of the blast-like cells alongside the vascular structures, resembling the pDC-like AML phenotype component, whereas the majority of blasts resemble phenotypically characteristic BPDCN cells. **j** Two-dimensional density plots of average CpG site DNA methylation in BPDCN *vs* AML, CMML, t-ALL, and melanoma (low density: orchid; high density: yellow/orange). **k** Average genome-wide DNA methylation level (beta values) of BPDCN, AML, CMML, t-ALL, and melanoma; for BPDCN subcluster estimates are shown as well. **l** DNA-methylation-based mitotic clock (epiCMIT) estimates for each entity and BPDCN subtypes. **m** Network enrichment against REACTOME for DMRs between BPDCN and AML; fold changes are color-scaled (red: higher DNA methylation in BPDCN; blue: higher DNA methylation in AML) and gene sets are denoted by light-brown nodes. **n** Pathway enrichment against REACTOME gene sets of gene-associated CpGs between BPDCN (blue) and AML (orange). **o** Pathway enrichment against REACTOME gene sets of promotor-associated CpGs between BPDCN (blue) and AML (orange). If not stated differently, differences between BPDCN and the four other entities were assessed by unpaired Wilcoxon test, and significant levels are indicated by asterisks (*p < 0.05, **p < 0.01, and ***p < 0.001).

To assess the potential of DNA methylation analysis in the differential diagnosis between BPDCN and neighboring entities, we performed a comparative analysis of DNA methylation data between our cohort and the BEAT-AML cohort as well as a CMML cohort previously published by Palomo et al. This analysis revealed a separation of samples into entity-specific DNA methylation classes in both an unsupervised (PCA; Fig. 2a) as well as a supervised (PLS-DA; Fig. 2b) approach. Distinction from T-cell acute lymphoblastic leukemia (T-ALL) and malignant melanoma, which was added for comparison, as another entity driven by a UV-related mutational signature, was even more pronounced (Fig. 2j). Intriguingly, we observed four borderline cases (Fig. 2c; BPDCN_01; 15; 27; 37), exhibiting a relevant amount of overlap between both groups in terms of DNA methylation. In contrast to the overall distinction between BPDCN and AML, we found three of these cases to exhibit synchronous concurrent manifestations of AML and/or transformed CMML with pDC features and one of these three cases even presented with genetic features typically encountered in AML with pDC-features (*RUNX1* mutation; Fig. 2c, d–i). The patient was allocated closest to the 95% CI cut-off for the AML definition and exhibited an atypical, more immature C2-BPDCN phenotype. Intriguingly, the *RUNX1* mutation was observed at an approx. VAF of 50%, despite the obvious phenotypical heterogeneity with typical BPDCN, infiltrates alongside cells more typical of pDC-like AML emerging from an MDS, suggesting a partially shared clonal architecture. While RUNX1 mutations are considered rather rare in BPDCN we observed eleven cases, however, most of these (nine out of eleven) where these mutations were subclonal events and some cases even exhibited syn- and/or metachronous myeloid neoplasms other than BPDCN. Subsequent comparative pathway enrichment analysis against HALLMARK and REACTOME gene sets for most DMRs within gene-body regions revealed a significant enrichment across RHO GTPases, cell migration control, and leukemic stem cell maintenance (HSF1 activation) in BPDCN, whereas promotor regions in BPDCN compared to AML were methylated to a significantly higher degree in epigenetic and transcriptional regulation as well as TP53 regulation and cell cycle control. (Fig. 2m–o).

### The BPDCN genome is characterized by an exceptional degree of DNA methylation loss and epigenetic signs of mitotic stress

Global DNA methylation is commonly diminished in malignant compared to healthy cells, being stably maintained in the latter. In this line, BPDCN revealed the highest methylation loss compared to AML, CMML, T-ALL, and melanoma irrespective of the C1 or C2 subtype (Wilcoxon rank-sum test, *p* < 0.001) (Fig. 2k) [8, 28–31]. Intriguingly, the aggressive clinical nature of BPDCN was further reflected in our epiCMIT analysis, as a DNA methylation-based mitotic clock, which recapitulates the proliferative history of a given tumor sample [32]. epiCMIT predicted unveiled an accelerated mitotic history in BPDCN compared to CMML and even AML, matched only by T-ALL (Fig. 2l). Although an independent prognostic impact of a high epiCMIT score was observed for a wide range of blood cancers, we observed no such trend in our cohort, plausibly attributable to the limited sample size.

### DNA methylation of tumor suppressor genes is highly deregulated in BPDCN compared to CMML and AML

**Fig. 3:**
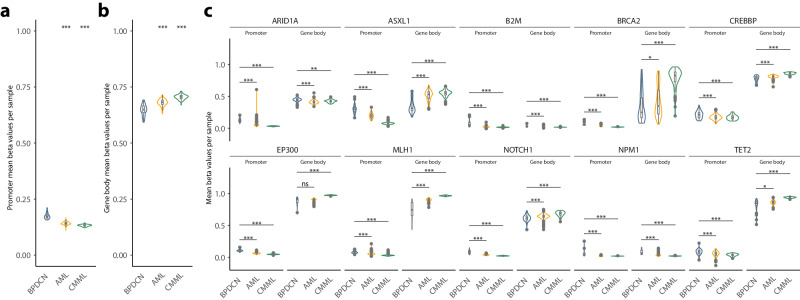
DNA methylation of tumor suppressor genes in BPDCN, AML, and CMML. **a** Average promoter DNA methylation (beta values) of tumor suppressor genes in BPDCN, AML, and CMML. **b** Average gene body DNA methylation (beta values) of tumor suppressor genes in BPDCN, AML, and CMML. **c** Average promoter and gene body DNA methylation in selected tumor suppressor genes (for a complete representation of significantly divergent TSGs see Supplementary Fig. 2). Differences between BPDCN and the AML/CMML were assessed by unpaired Wilcoxon test and significant levels are indicated by asterisks (*p < 0.05, **p < 0.01, and ***p < 0.001).

In keeping with a substantially deregulated DNA methylation profile in BPDCN and global loss of DNA methylation, we observed promotor regions of tumor suppressor genes to be methylated to an exceptionally high degree, compared to CMML and AML (Fig. 3a). A significantly lower level of gene-body associated methylation in the same genes was identified (Fig. 3b), indicating a substantial oncogenic impact of deregulated DNA methylation in BPDCN (manually selected candidate TSGs, Fig. 3c, for exhaustive information on deregulated TSGs see Supplementary Fig. 2). Strikingly, we hereby observe an increasingly deregulated DNA methylation profile from CMML to BPDCN in parallel to the aggressiveness of the entity’s clinical behavior.

### DNA methylation patterns and gene expression signatures differentiate C1 and C2 subtypes in BPDCN and shape a JAK/STAT-driven profile in C2-BPDCN

**Fig. 4:**
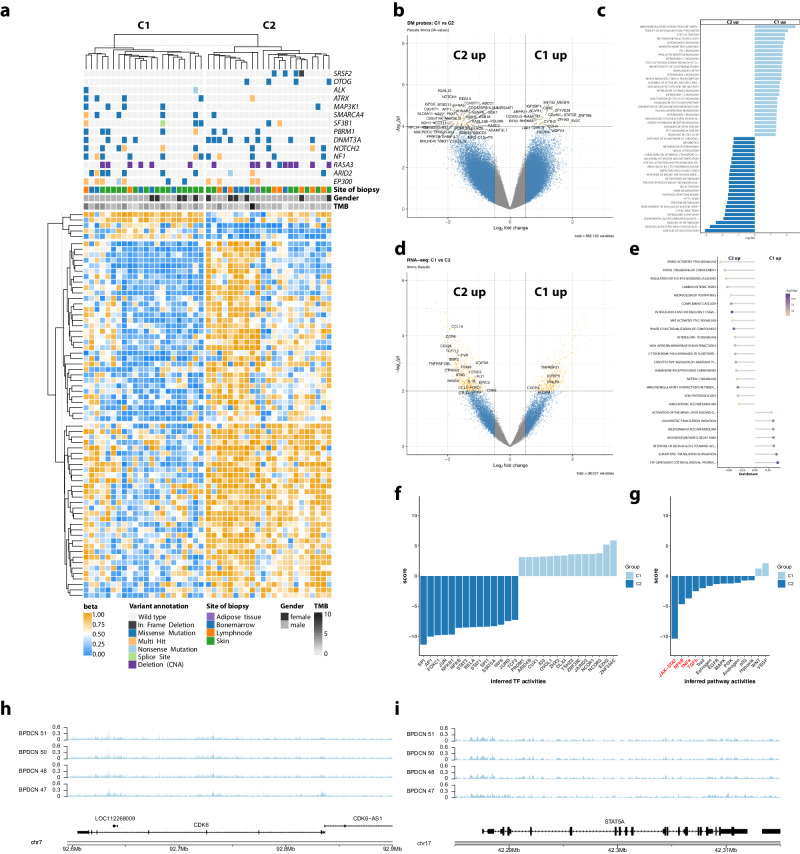
Differential DNA methylation, expression, and mutation patterns in BPDCN subtypes C1 and C2. **a** Top part of the heatmap shows mutational patterns of 14 genes previously identified as significantly enriched between the two clusters [6]. The bottom part shows beta values (DNA methylation levels) of CpG sites where an absolute mean difference above 0.25 was observed between C1 and C2. **b** Volcano plot of beta values showing log_2_ fold-changes and *p* values with gene annotations for significantly different CpGs (*p* < 0.0001). **c** Gene set enrichment analysis results of DNA methylation data against REACTOME gene sets (*p* < 0.05). **d** Volcano plot of expression profiles showing log_2_ fold-changes and *p* values with gene annotations for significant differentially expressed tumor suppressors genes, oncogenes, and genes involved in cell adhesion/cell cycle (*p* < 0.0001). **e** Gene set enrichment analysis of RNA-seq data against REACTOME gene sets (*p*_adj_ < 0.001, absolute enrichment >0.3). **f** Transcription factors with significantly different inferred activity (*p* < 0.05) in C1 (light blue) and C2 (dark blue). **g** Pathway activities in C1 (light blue) and C2 (dark blue) with significantly different pathway activities shown in red font (*p* < 0.05). **h**, **i** FFPE-ATAC-seq estimates of chromatin accessibility for *CDK6* and *STAT5A* for four BPDCN cases belonging to subcluster C1. CDK6 is located on the minus strand and *STAT5B* on the plus strand, respectively.

Building on transcriptional BPDCN subtype classification, we performed a differential DNA methylation analysis. Alongside differentially mutated genes in BPDCN, we discovered 114 probes, which were differentially methylated between C1 and C2 (*p* < 0.0001; 10,136 probes with *p* < 0.01), corresponding to a relatively similar methylome, in keeping with our above PCA/PLS-DA (depicted as scales beta values in Fig. 4a; beta values see Supplementary Table 4).

To gain insight into the effect of differential DNA methylation of both subtypes, we assigned differentially methylated probes to their respective genes (Fig. 4b). GST (Fig. 4c) revealed enrichment in elevated DNA methylation levels for interleukin signaling genes and prominent members of JAK/STAT signaling (STAT5B) in C1-BPDCN, whereas C2-BPDCN samples exhibit significantly pronounced DNA methylation of posttranslational modifications and metabolic processes (e.g., vitamin and heme metabolism).

Next, we performed differential gene expression analysis (Fig. 4d, e). Hereby, we observed an induction of innate and adaptive immunological processes alongside an upregulation of extracellular matrix interactions in C2 cases. Further, we found several prominent candidate genes, including *STAT5A*, *CDK6*, *CCR4*, *CCND2,* and *FOXO1* to be expressed at significantly higher levels in C2 cases. Corresponding to higher gene expressions in C2, *CDK6* (*p* = 5.3 × 10^−4^, log_2_ fold-change 1.52) and *STAT5A* (*p* = 5.5 × 10^−3^, log_2_ fold-change 1.19) had promotor-associated sites that were significantly higher methylated in C1-BPDCN leading to relative transcriptional inactivation in C1. Correlation of DNA methylation at promotor and gene body sites and relative RNA-seq-derived gene expression data yielded a substantial number of significant correlations. Findings regarding the enrichment of gene promotor methylation in particular TSGs are only partially recapitulated on the transcriptional level (Supplementary Table 5 and Supplementary Fig. 4a). Various statistically significant correlations between DNA-methylation at either type of site and gene expression, regardless of potential confounding factors, including copy number variants and mutations, affecting prominent TSGs and oncogenes are identified through both a focused and genome-wide approach (see Supplementary Fig. 4b, c). Significantly correlated candidates include *BCL2*, *ALK*, *GNAQ*, and *RUNX1*.

To further focus our observations on potential therapeutic applicability, we inferred TF activities from bulk RNA-seq data employing CollecTRI as a resource for TF pathway activity inference with PROGENy. Hereby we observed the transcriptional mirror image of the divergence in DNA methylation profiles between C1 and C2-BPDCN. In particular, we observed significantly higher activity of NFkB (driven by FOXC1, NFKB1, and NFKB; *p* < 0.05) and more strikingly JAK-STAT (predominantly driven by STAT3, STAT1, and STAT5A; *p* < 0.05) associated TF in C2 and an EZH2 dependence in C1-BPDCN (Fig. 4f, g). We supplemented these observations by FFPE-ATAC-seq of four C2-BPDCN, which revealed substantial chromatin accessibility, in keeping with our epigenetic and transcriptional findings (Fig. 4h, i).

### Tumor microenvironment by MethylCIBERSORT and immunohistochemistry reveals distinct immunological subtypes correlated with tumor genomics

**Fig. 5:**
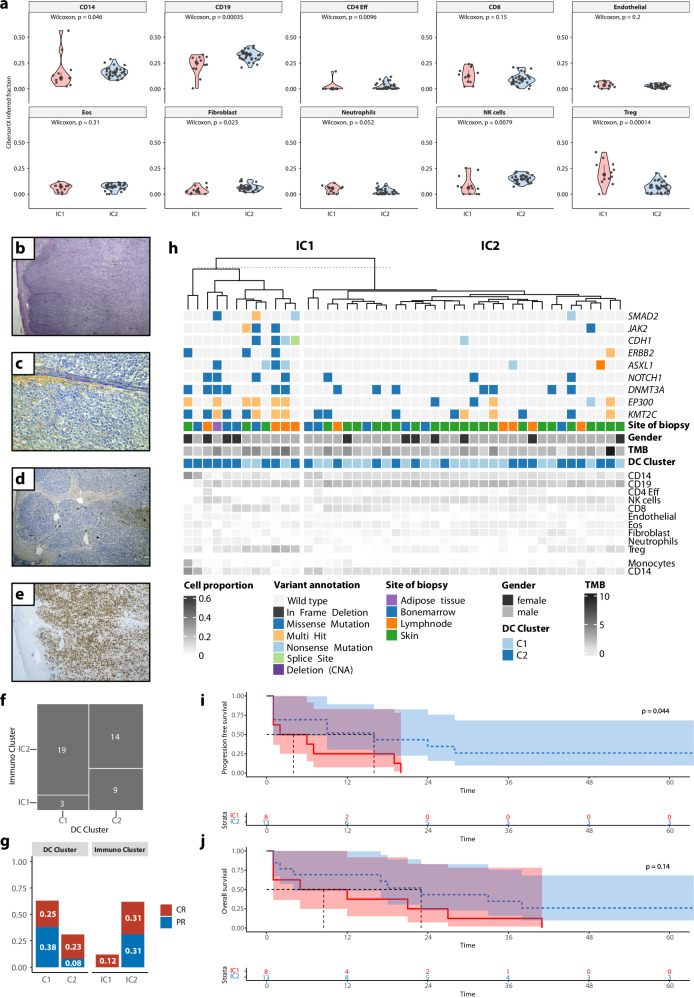
Tumor immune composition by MethylCIBERSORT identifies clusters of divergent immunogenicity. **a** DNA methylation data were deconvoluted according to immune cell populations (MethylCIBERSORT). This revealed two different types of BPDCN (Treg and CD14 driven; named IC1 and IC2, respectively) presenting with significantly differing immune cell subsets in regard to the markers CD14 (monocytes/macrophages), CD19 (B-cells), CD4 (T-helper cells) as well as fibroblasts, NK-cells and T-regulatory cells (T-regs). **b**–**e** A borderline BPDCN/AML pDC-like case analyzed by MethylCIBERSORT and a comparative immunohistochemical assessment of the tumor microenvironment is presented. **b** H&E staining reveals a cutaneous infiltrate covered by an intact epidermis. **c** Giemsa staining reveals small blastoid cells with partly roundish occasionally monocytoid nuclei, small nucleoli, and weakly basophilic cytoplasm with increased mitotic activity. **d** Staining for myeloid peroxidase reveals expected negativity in the malignant infiltrate alongside a few positive, tumor-infiltrating myeloid cells. **e** However, CD14-Expression highlights both a typical negative BPDCN population, as well as a relevant monocytoid population, including few tumor-infiltrating monocytes alongside a larger subgroup of malignant cells. **f** Mosaic plot visualizing the overlap between transcriptional phenotype C1/C2 and IC1/IC2. **g** Overall, complete and partial response rates according to C1/C2 and IC1/IC2. **h** Oncoplot displays mutational patterns of 9 genes that were found to be more differentially mutated between IC1 and IC2. Additionally, the heatmap illustrates TME cell proportions for each individual sample. **i**, **j** Progression-free (PFS) and Overall survival (OS) analysis for patients with available clinical follow-up according to IC1 vs IC2 identifies a significant inferior prognostic impact for the IC1 subtype regarding PFS accompanied by a trend towards inferior OS.

To evaluate the TME, we conducted a MethylCIBERSORT analysis on all 45 high-quality genome-wide DNA methylation profiles. From inferred relative abundance of T-cell subpopulations, B-cells, natural killer (NK)-cells neutrophils, monocytes, eosinophils, and stromal cells two TME classes (IC1 and IC2) were predicted. The smaller class (IC1) exhibited a depletion in monocytes, B- and NK-cells, an enrichment in Tregs, and a trend towards higher counts of neutrophils and cytotoxic T cells (Fig. 5a). Additional deconvolution revealed a significantly higher proportion of keratinocytes in skin samples compared to non-skin samples (*p* = 0.036). These observations were subsequently validated via IHC for tumor-infiltrating T cells and monocytes with a significant correlation between relative distributions of cell populations (Fig. 5b–g; monocytes: Pearson correlation coefficient = 0.6437, *p* = 1.84 × 10^−6^ and T-cell populations: Pearson correlation coefficient = 0.3809 *p* = 0.0098). The correlation of TME with mutational profiles (restricted to the eight genes mutated in at least 30% of BPDCN cases) is presented in Supplementary Fig. 5. We observed enrichment in mutations affecting *CDH11*, *ERBB2*, *ASXL1, EP300, KMT2C, JAK2, SMAD2, NOTCH1*, and *DNMT3A* (Fig. 5h, Supplementary Fig. 6a) and a trend towards a higher TMB (Supplementary Fig. 6b) in IC1. Further, IC1 patients had significantly shorter progression-free survival (*p* = 0.044) and a trend towards inferior OS (*p* = 0.14) (Fig. 5i, j). Similar characteristics in terms of TME composition were observed by immunohistochemistry and MethylCibersort with enrichment of T-cells in IC1 and monocytes in IC2 (Supplementary Fig. 6c–f). epiCMIT was applied to estimate the history of proliferative stress/DNAm age in both immunological clusters and revealed significantly higher proliferative stress in IC2 compared to IC1, which resembles DNA methylation-based pre-aging in this subgroup (*p* = 0.038; Supplementary Fig. 6g). A partial overlap between TME classes IC1/2 and transcriptional clusters C1/C2 with a strong enrichment of IC2 cases in C1 BPDCN patients was observed. An integrated summary of immunohistochemical and molecular features including cluster allocation is provided in Supplementary Table 3. Of particular clinical relevance and in keeping with the impact on survival we identify substantial differences in terms of overall response rates between C1/C2 (66% vs 31%) and IC1/IC2 (12% vs 62%) (Fig. 5f, g). Previous reports identified a correlation between DNA methylation and chronological age [33, 34]. We observed a trend towards higher levels of tumor-infiltrating T-cells in C2-BPDCN samples despite the overall higher TMB in C1 patients, regardless of patient age or location of the tumor sample by both MethylCIBERSORT and IHC (Supplementary Fig. 7a, b).

### DNA methylation-based clusters cannot be fully recapitulated in transcriptional BPDCN subtypes

The most 5600 variable CpG probes were subject to discovering DNA methylation subtypes using K-means clustering. The optimal number of clusters was determined using the average silhouette method and gap statistics. Both methods agreed on the optimal number of clusters (*n* = 2). Upon unsupervised cluster analysis, we observed no statistically significant recapitulation of transcriptional BPDCN subtypes C1 and C2. Some overlap was, however, found in shared genomic features (*EP300* and *ATRX* mutations in C1 and MethC1), consistent with higher TMB in C1 and MethC1. A significantly more pronounced overlap was apparent upon correlation analysis between DNA methylation-based cluster allocation and immunological clusters according to our methyCIBERSORT-derived immunoclusters (Supplementary Fig. 8a, b).

### Mutational drivers and promotor status of epigenetic regulators shape the proliferative fate and DNA methylation profile of BPDCN

**Fig. 6:**
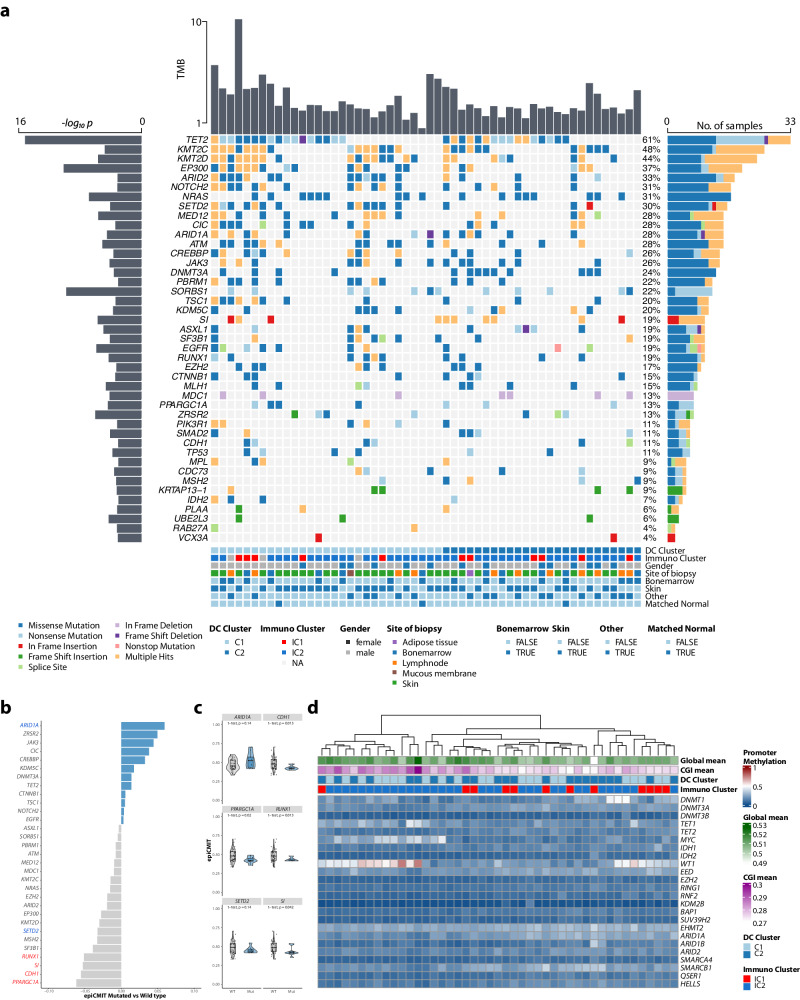
Impact of significant driver mutations and DNA methylation status of epigenetic regulators on proliferative history and global CGI DNA methylation. **a** Oncoplot displaying putative driver genes inferred by MutSigCV analysis in addition to tumor mutational burden (TMB; upper bar plot), *−log*_*10*_ *p* values (left bars), and the number of samples harboring mutations in a given gene (right bar). In total 6065 bioinformatically deleterious mutations, in 3970 genes were observed. Of these indels and SNVs, 4999 were missense (82.4%), 321 nonsense (5.3%), 12 non-stop (0.2%), and 487 indel mutations (8.0%). Mutation types are color-coded, and covariates are shown below for each sample (covariate “Other” refers to samples with tissue affected other than skin or bone marrow). **b** Analysis of which significant mutational driver alterations may confer a higher or lower proliferative capacity to BPDCN cells. Statistically significant associations are delineated in red and blue, respectively. **c** Comparative analysis regarding proliferative capacity in selected genes’ mutant and wild-type status. **d** A heatmap depicting the DNA methylation status of gene promoter regions across a set of preselected epigenetic regulators associated directly and indirectly with global DNA methylation, as described [48]. Average global DNA methylation and CpG islands (CGI) are estimated per patient.

To provide genomic context for DNA methylation profiles obtained within this study, we performed complementary WES on all patients, who were not part of our previous molecular BPDCN study (Fig. 6a). WES confirmed a higher mutational load in C1 BPDCN (Supplementary Fig. 3b), further supporting a non-mutational mechanism driving C2-BPDCN such as the deregulated DNA methylation and transcriptional profile outlined above. The pathophysiological role of epigenetic features within the C2 cluster is supported by more frequent *DNMT3A* (14% vs 36%) alterations bordering on statistical significance in this limited cohort and the significant mutational enrichment for splicing gene *SRSF2* (0% vs 16%; Supplementary Fig. 3a).

Further, we wondered whether specific mutational drivers were associated with the proliferative history of a given case, thereby integrating mutational and epigenetic datasets to identify genetic alterations, which could confer a selective advantage as mitotic accelerators. We observed a trend towards a proliferative increase in cases harboring *ARID1A*, *ZRSR2*, *JAK3*, *CIC,* and *CREBBP* mutations and a (significant) decrease in mitotic activity in cases with mutant *PPARGC1A*, *CDH1*, *SI*, *RUNX1*, *SF3B1*, *MSH2* and *SETD2* (Fig. 6b, c). In summary, our findings suggest, that the proliferative potential of BPDCN is determined by mutational driver events. To unravel additional determinants, that shape the globally demethylated landscape in BPDCN, we investigated the promotor DNA methylation status of a set of genes associated with epigenetic regulation (Fig. 6d). Hypermethylation of the promotor region (promoter CGI methylation >0.2) of *DNMT1*, *WT1*, *MYC*, and *TET1* was hereby observed in 42.2%, 73.2%, 40.0%, and 53.3% of patients, respectively. Further, we observed a significant correlation between promotor methylation and overall CGI methylation for 22 of the 24 genes (Spearman’s rank correlation, *p*_adj_ < 0.05) signifying a substantial impact on the epigenetic landscape in BPDCN. Only DNMT1 and KMD2B did not show a significant correlation. Of note, a substantial overlap between patients harboring *WT1* and *MYC* promoter methylation was observed in 16/45 cases (35.6%). Given that *MYC* constitutes a known *WT1* target, this hints at a coupled mechanism in BPDCN pathogenesis [35] (Fig. 6b, c).

## Discussion

BPDCN is a heterogeneous disease that poses a multi-level challenge in terms of diagnostics and treatment alike. Unraveling the epigenetic characteristics of the disease may aid in providing a correct and timely diagnosis, which is crucial for the initiation of specific treatments. Through comparative analysis of the DNA methylation landscape of BPDCN, AML, CMML, and T-ALL, we identify distinguishing features and provide unprecedented insights into the molecular pathogenesis of BPDCN, which may in turn facilitate the development of more refined therapies. In this integrated molecular study we report on the largest BPDCN cohort studied so far and systematically defined genomic and transcriptional signatures in the context of genome-wide DNA methylation [4, 8, 10, 36–42]. Hereby, we made three essential observations.

First, DNA methylation profiles allowed for a clear distinction between BPDCN, AML, CMML, and T-ALL. BPDCN is characterized by global demethylation and merely localized DNA hypermethylation showing dominant signs of mitotic stress, resembling a pronounced variant of the canonical cancer methylome. Several patients, for whom a borderline DNA methylation profile between AML and BPDCN was identified, were found to resemble cases with either syn- or metachronous development of CMML/AML or a molecular and phenotypical constellation resembling AML with pDC-like features [9, 10]. In one case, we observed the simultaneous occurrence of typical BPDCN and pDC-AML infiltrates, the latter seemingly emerging from an underlying MDS. Moreover, all but one of these cases exhibit the more immature C2-BPDCN transcriptional profile [6]. This aligns with observations in B-cell malignancies and solid tumors where the DNA methylation profile reflects the degree of maturation of the cell-of-origin [32, 43]. In keeping with previous observations, we observed syn- or metachronous myeloid non-BPDCN neoplasms in a substantial subgroup of the cohort and quite intriguingly *RUNX1* mutations with VAFs suggestive of subclonal aberrations in several cases. Recently, clonal hematopoiesis (CH), disrupting epigenetic regulators in the majority of BPDCN cases, was proposed as an underlying mechanism rendering mutations in RAS signaling (*NRAS*, *KRAS*) and tumor suppressors like *TP53* and *ATM* secondary clonal events and thereby more specific in BPDCN pathogenesis [44]. Our previous genomic studies revealed a significant overlap in mutational drivers between BPDCN and the abovementioned entities, underscoring the close molecular relatedness between BPDCN and especially AML/CMML, which was recently further illustrated in cases of divergent clonal evolution from a CHiP constellation. This might be particularly relevant in cases with syn-/metachronous AML/CMML and/or (subclonal) *RUNX1* mutations, as observed in a substantial subgroup of our cohort. The present study, however, revealed a significant impact on signaling processes shaping the specific phenotype of BPDCN in particular. This can therefore be harnessed for diagnostics beyond established approaches [20, 28, 45]. DNA methylation profiling revealed a canonical epigenetic deregulation of TSGs with putative oncogenic effects, vastly exceeding previous observations in related entities [28, 29]. Intriguingly, we observed several mutational drivers of mitotic stress, signified through a proliferative epigenetic signature. In line with previous reports, we found *ARID1A* mutant BPDCN samples to exhibit pronounced signs of proliferative activity, while decreased mitotic activity in *RUNX1* mutant BPDCN is in keeping with reduced proliferation in hematopoietic stem cells harboring similar mutations [46, 47]. At the same time, unsupervised clustering of genome-wide DNA methylation levels only partially recapitulated transcriptional clusters, which was, however, previously observed in other blood cancers (T-ALL) as well [48].

Second, recently established molecular subgroups of BPDCN differ in terms of both transcriptional profile by RNA-seq and to a lesser extent by DNA methylation. Expanding on previous studies, we found predominant pathways deregulated by these circumstances to include innate and adaptive immunological processes, for which gene expression was significantly induced in C2-BPDCN. This corresponds to elevated DNA methylation levels in interleukin/inflammatory signaling genes in C1-BPDCN, leading to a relative upregulation of the interleukin 4/13 interactome in C2-BPDCN [49]. Interrogating our bulk RNA-seq data for TF activity, we observed the transcriptional mirror image of the divergence in DNA methylation profiles between C1 and C2-BPDCN. Regarding potential therapeutic targets, we observed significantly higher JAK-STAT- (predominantly driven by *STAT3*, *STAT1*, and *STAT5A*) and NFkB- (driven by *FOXC1*, *NFKB1*, and *NFKB*) associated TF activity in C2 in contrast to an *EZH2*-dependence in C1-BPDCN. Potent inhibitors for molecularly informed therapeutic combinations with tagraxofusp are readily available [50]. These observations were then verified by FFPE-ATAC-seq, revealing substantial chromatin accessibility at highly expressed loci, including *CDK6* and *STAT5A*, in keeping with our epigenetic and transcriptional findings.

Finally, we gained insight into the TME through the combined analysis of MethylCIBERSORT and immunohistochemistry. There are two, prognostically relevant immunologic classes (IC1 and IC2) of BPDCN, an unfavorable IC1 subgroup with lower PFS and ORR (comprising ~25% of patients) harboring higher TMB and significant enrichment for *ERBB2*, *ASXL1, EP300*, and *KMT2C* mutations alongside a TME relatively depleted of NK-cells, monocytes, and B-cells but enriched in Tregs while IC2 showed pronounced monocyte, B- and NK-cell infiltrates and superior ORR and PFS. Beyond PD-L1 expression levels, our comprehensive MethylCIBERSORT approach identified a subset of immunologic hot cases in which immunotherapeutic strategies beyond tagraxofusp seem to be promising [51]. Moreover, we found that IC2 had a significantly higher epigenetic age but there was no difference regarding chronological age between both subgroups, congruent with previous observations in NSCLC [23], where a higher epigenetic age was able to emulate the malignant potential of tumors with a high mutational load or decisive driver mutations including *TP53* [23]. In addition to the immunologically defined classes, we observed an elevated level of TILs in atypical C2-BPDCN. Our future goal is now to dissect the TME in even greater detail by single-cell RNA sequencing and spatial transcriptomics. Relatively high rates of positivity for B-cell antigen CD79a and myeloid antigen CD33 compared to large-scale flow cytometry studies are partially attributable to the TME, which will most likely be more precisely dissected in multi-parameter follow-up analyses at single-cell resolution [7, 12].

Limitations of the current study include a restricted number of cases, sampled at various anatomical sites, alongside a partial lack of information including clinical follow-up. In addition, the limitations of the Illumina EPIC array, chosen for comparability with large-scale studies on neighboring entities, are acknowledged in that it is based on a bisulfite conversion and cannot distinguish between 5-methylcytosine, 5-hydroxymethylcytosine, 5-formylcytosine, and 5-carboxycytosine. Although the levels of total 5hmC detected across the human genome in both healthy and cancer cells are ~14-fold lower than those of 5mC, indicating that most observations in this study indeed stem from 5mC, we will partially address this through a coupled analysis of a split aliquot by both conventional bisulfite conversion as well as the TET-assisted bisulfite approach in a follow-up study including longitudinal sample collection [52]. Moreover, a substantial subset of tagraxofusp-treated patients in the context of routine clinical care alongside patients treated with novel agents including IMGN632, BCL2 inhibitors as well as allogenic or autologous CAR-T cell therapies or combination approaches within the scope of clinical trials will make a valuable addition to future studies.

In conclusion, we can reliably distinguish BPDCN from its related entities. Further, we unravel the epigenetic and transcriptional underpinnings of the two recently defined subtypes of BPDCN, identifying divergent potential targetable vulnerabilities and characterizing immunologically and prognostically meaningful BPDCN.

### Supplementary information

Materials and Methods

Supplementary Table 1

Supplementary Table 2

Supplementary Table 3

Supplementary Table 4

Supplementary Table 5

## Data Availability

Raw data for BPDCN samples have been downloaded from the European Genome-phenome archive (EGA) under the previous accession number EGAS00001006166, additional cases were deposited under accession number EGAS00001007201, respectively. EPIC array data have been deposited in Gene Expression Omnibus (GEO) under accession number GSE230487.
