# Supplementary material for: Genome-wide DNA methylation-analysis of blastic plasmacytoid dendritic cell neoplasm identifies distinct molecular features
Source: Leukemia. 2024 Apr 10;38(5):1086–98. doi: 10.1038/s41375-024-02240-8 (PMC11073989; doi:10.1038/s41375-024-02240-8)
Supplement: Supplementary file 1 — Materials and Methods [file 41375_2024_2240_MOESM1_ESM.pdf]

Supplementary Materials and Methods.

Case selection, clinicopathological assessment, whole exome, and whole transcriptome sequencing

For this retrospective analysis, we reviewed our institutional archive for cases of histologically confirmed BPDCN between January 2001 and April 2022. The study was approved by the ethics committee of the University of Lübeck (reference-no 18-311) and conducted in accordance with the declaration of Helsinki. Patients at the Consultation Center for Hematopathology provided written informed consent regarding routine diagnostic and academic assessment, including genomic studies. Histopathological work-up was performed as described[1] and yielded 74 cases of BPDCN, 54 of which had sufficient FFPE tissue samples available for subsequent molecular analysis were selected and subjected to a comprehensive immunophenotypic workup (47 samples were included in a previously published investigation of the mutational landscape in BPDCN[1]). Genomic DNA and RNA were extracted from three 5µm FFPE tissue sections of either tumor or normal tissue (where available; n = 3) employing Maxwell® RSC DNA FFPE kit and Maxwell® RSC RNA FFPE kit (both Promega). WES and RNA-Seq following library preparation using Agilent SureSelect Human All Exon V6 library preparation kit (Agilent Technologies) and NEBNext® UltraT Directional RNA Library Prep Kit (New England BioLabs), respectively were performed on a NovaSeq platform (Illumina) at Novogene (UK) Co. as described [2]. Tumor whole exome libraries were sequenced to a median depth of 131x (mean 134 ± 52 s.d.) and normal libraries reached a median depth of 67x (mean 83 ± 36 s.d.).

Whole exome data processing and variant calling

Sequencing data from WES was processed using the same workflow as previously described [1]. Briefly, raw reads (paired-end fastq format) were trimmed (adapter and quality values) applying FASTP (v0.23.0; minimum length, 50 bp; maximum unqualified bases, 30%; trim tail set to 1)[3];

trimmed reads were mapped to GRCh38 using bwa mem (v0.7.15)[4] and mappings were converted into BAM format using PICARD TOOLS (v2.18.4). Next, mate-pair information was fixed, PCR duplicates were removed, and base quality recalibration was performed using PICARD TOOLS, GATK (v4.2.3.0)[5] and dbSNP v138 [6]. Single nucleotide variants (SNVs) and short insertions and deletions (indels) were identified following GATKs best practices for somatic mutation calling (matched normal-tumor mode for samples with normal tissue available (n=3) and tumor-only mode for samples without normal tissue available). MUTECT2 (GATK)[7] was applied to the processed mappings with GNOMAD variants as germline resource and the b38 exome panel from the 1000 genome project as a panel of normal, capturing the expected repertoire of germline variants to be expected in a Central European study population. Next, FFPE read orientation artifacts were identified and removed according to GATK guidelines. Filtered variants were annotated using VARIANT EFFECT PREDICTOR[8] (VEP v103, GRCh38; adding CADD v1.6, dbNSFP v4.1a23, and GNOMAD r3.0 as additional resources) and annotations were converted into *MAF* format using VCF2MAF (V1.6.21) (DOI:10.5281/ZENODO.593251); coverage was extracted directly from the vcf INFO field. The top 20 frequently mutated genes (FLAGS)[9] were removed from further analysis and the remaining somatic variants were filtered as follows: minimum coverage of 40, minimum alternative allele coverage of 5, minimum variant allele frequency of 10%, and only variants with a frequency $< 0.1\%$ in 1000 genomes, GNOMAD, or ExAC were considered for subsequent downstream analysis. High-impact variants (CADD score > 10) in tumor suppressors according to Vogelstein et al. [10] were filtered as such that minimum coverage of 20 minimum, minimum alternative coverage, and minimum variant allele frequency of 10% was required. Genes mutated more often than expected were identified by applying MUTSIGCV (v1.41)[11] and potential drivers were identified using $p < 0.001$. Tumor mutational burden (TMB) did not differ between tumor-only samples and samples with matched normal tissue at the finalization of the filtering process (Wilcox test, $p = 0.1004$).

Transcriptome Data Processing, Quantification, Deconvolution, and Analysis

Gene expression sequencing data were processed in the same manner as described previously [1]. Briefly, gene expression profiles were retrieved from adapter trimmed reads (FASTP as described above) using STAR ALIGNER (v2.7.4b)[12] against GRCh38 (GENCODE v37) as reference. On average 73.5 million reads (median 81.1) were successfully mapped to the human reference per tumor sample and 29.6 million reads (median 30.4) per normal sample, respectively. Count profiles were normalized applying MIXNORM (v0.0.0.9000; 50 iterations, tolerance set to 0.1) [13], which removes unwanted biological and technical effects from FFPE material that can bias the signal of interest. Differentially expressed genes on normalized expression values between two conditions were identified using a linear modeling approach (LIMMA package, v3.50.1)[14].

Pathway enrichment analysis against REACTOME gene sets (MSIGDF R package v7.4) on significantly differentially expressed genes was performed using a rank-MANOVA-based approach as implemented in MITCH (v1.12.0; priority on significance) [15]; NF-$\kappa\beta$ pathway (KEGG) was added manually to the REACTOME gene set.

Previously published scRNA-data from Villani et al.[16] was used to infer the cell-type composition of the bulk RNA-Seq data with respect to dendritic cells and monocytes by applying a deconvolution of damped weighted least squares (DWLS) method as described previously [1].

Transcription factor and Pathway activities from RNA-seq data

Transcription factor activities were inferred from RNA-seq data using prior knowledge as provided by Collection of Transcriptional Regulatory Interactions (CollecTRI (Preprint at https://doi.org/10.1101/2023.03.30.534849 (2023)), DECOUPLER v2.6.0), which provide a curated collection of 1,175 transcription factors. Briefly, t-values between two conditions (BPDCN DC cluster) were estimated using LIMMA. Transcription factor activities were

estimated on *t*-values using weighted means and activities with $p < 0.05$ were plotted (28 transcription factors).

Pathway activities for curated pathways with weights for each interaction were estimated using Pathway RespOnsive GENes for activity inference (PROGENy [17], gene signatures for 14 pathways) on limma estimated *t*-values (as above).

Genome-wide DNA methylation profiling and data analysis

Whole-genome DNA methylation analysis was carried out on all 54 cases of the study cohort employing the Illumina EPIC array at ATLAS Biolabs. Bioconductor R package MINIFI (v1.46.0) was used to further process raw IDATs that were previously generated from iScan. The quality of samples was checked by using mean detection *P*-values and only samples with *P*-values <0.05 were kept for further processing (five samples excluded). In addition, according to in-house bioinformatic QC pipelines (Glaser *et al.*, in preparation) was applied (one additional case was excluded). The remaining samples were normalized using quantile normalization (funtion *preprocessQuantile*) and DNA methylation data predicted sex was compared to the actual sex. Samples, where the predicted sex did not match with the actual sex, were removed (four samples removed), leaving 45 samples for further analysis. DNA methylation probes were quality filtered and probes with non-significant *P*-values were removed ($P > 0.01$). Additionally, cross-reactive probes and BOWTIE2 multi-mapped probes were removed, and M- and beta-values of the remaining probes were extracted [18].

Differentially methylated probes between two conditions were identified using a linear modeling approach as implemented in LIMMA. Generalized gene set testing on differentially methylated probes was performed by applying the *gsameth* function (MISSMETHYL package v1.34.0) against the REACTOME and/or HALLMARK gene sets (MSigDB v7.5).

Comparative analysis with genome-wide DNA methylation data from different cell types

To assess epigenetic differences between BPDCN and several cell types, we used data from different sources. Raw DNA methylation data (IDAT files) from B-lymphoid cells (n = 23), naïve CD4+ (n = 23), naïve CD8+ cells (n = 19), granulocytes (n = 10), monocytes (n = 24) and NK-cells (n = 20) was retrieved from GEO accession GSE184269 [19]. Data were processed as described above with the exception that normalization was performed using subset-quantile within array normalization (*SWAN*, MISSMETHYL) and beta-values were extracted. DNA methylation profiles (beta-values) for dendritic cells (n = 6; GEO accession GSE71837[20]) and hematopoietic stem cells (n = 5; GEO accession GSE63409[21]) were downloaded using GEOquery (v2.68.0).

Data sets were combined (including 45 BPDCN samples), probes matching to chromosome X or Y were removed, and only probes present in all studies were to remove unwanted variation between the data sets (batch effects). Unwanted technical variation was removed using a two-stage approach (RUVm). First, a standard LIMMA analysis was performed to identify empirical control probes (ECPs). Next, the results from stage 1 were used to perform a second differential DNA methylation with RUV-4 (*RUVfit* function provided by the MISSMETHYL package) and adjusted beta-values were extracted. Region-level analysis was performed to call differentially methylated regions (DMRs) using adjusted beta-values between BPDCN and dendritic cells using DMRcate (v2.13.0) and gene set testing was performed on DMRs with fdr < 0.1 and absolute difference above 0.3 against REACTOME and HALLMARK gene sets using the *gsaregion* function (MISSMETHYL). Linkage of genes and enriched REACTOME pathways was performed for DMRs with fdr < 0.01 and absolute difference above 0.3 using *enrichPathway* (REACTOMEPA, v1.44.0, *q*-value < 0.1) and *cnetplot* (ENRICHPLOT, v1.20.0).

Comparative analysis with genome-wide DNA methylation data from acute myeloid leukemia

Regarding epigenetic discrepancies that may aid in the distinction of borderline cases between BPDCN and its predominant differential diagnosis, acute myeloid leukemia (AML), we comparatively analyzed our dataset with a comprehensive, previously published AML cohort, incorporating 243 samples for which genetic subtype and genome-wide DNA methylation data was available (GEO accession GSE159907[22]). Data (IDAT files) was processed (including 45 BPDCN samples) as described above using SWAN to normalize the data and probes from chromosome X or Y were removed. Unwanted variation was removed using a two-stage approach (RUVm). First, Illumina negative control (INCs) data for EPIC arrays was extracted (411 probes) and differential DNA methylation analysis using RUV-inverse with INCs as negative control features was performed. Next, the results from stage 1 were used to perform a second differential DNA methylation with RUV-4 and adjusted M-values were extracted for further analysis. Region-level analysis was performed to call differentially methylated regions (DMRs) using adjusted M-values between BPDCN and AML using DMRcate (v2.13.0) and gene set testing was performed on DMRs with fdr < 0.1 and absolute difference above 0.2 against REACTOME and HALLMARK gene sets using the *gsaregion* function (MISSMETHYL). Gene sets with $P < 0.01$ were considered as significantly enriched. Linkage of genes and enriched REACTOME pathways was performed for DMRs with fdr < 0.01 and absolute difference above 0.2 using *enrichPathway* (*q*-value < 0.1) and *cnetplot*.

Comparative analysis with genome-wide DNA methylation data from other entities

Raw data files (IDAT) from AML (n = 316; GEO accession GSE159907), T-cell acute lymphoblastic leukemia (t-ALL, n = 156; GEO accession GSE155339[23]), and melanoma (n = 450, TCGA project TCGA-SKCM) were downloaded and processed (including 45 BPDCN samples) as described above using SWAN to normalize the data; probes from chromosome X or Y were removed and beta-values were extracted. Additionally, beta-values for chronic myelomonocytic leukemia (CMML, n = 65; GEO accession GSE105420[24]) were downloaded

and merged with the processed data. Unwanted variation was removed using a 2-stage approach as described in the section above ('Comparative analysis with genome-wide DNA methylation data from different cell types').

Annotation of Methylated Regions

Probe annotations were extracted using the R package ILLUMINAHUMANMETHYLATIONEPICANNO.ILM10B4.HG19 (v0.6.0). Probes annotated as 'Island' were classified as CpG island (CGI) probes. Probes falling into 5' UTRs or within 1,500 bp of the transcription start site were classified as promotor sites; sites within gene bodies or 3'UTRs were defined as gene body sites.

Analysis of the tumor microenvironment by MethylCIBERSORT and immunohistochemistry

The cellular composition of the tumor microenvironment (TME) was assessed using MethylCIBERSORT as described. Beta values from raw IDATs and signature genes were deconvoluted according to immune cell populations [25]. Partitioning around medoids (PAM) was applied to identify immunologically hot versus cold immune tumors based on MethylCIBERSORT calculations and to assign the optimal cluster number in the data (testing from 2 to 10). Results obtained by MethylCIBERSORT were then validated through a correlative immunohistochemical assessment (IHC) of the T-cell and monocyte fraction of the TME. Antibodies and positivity cut-offs employed in the current study remain as described [1].

DNA-methylation-based mitotic clock estimation

To better comprehend the interaction between biological aging and molecular profiling in the light of the methylome, mitotic activity was estimated using epiCMIT. Mitotic clock was

estimated on batch-corrected beta-values for each sample and results were summarized per cohort [26, 27].

FFPE-ATAC-seq

ATAC-sequencing on FFPE tissue sections from four typical pDC-like BPDCN patients was performed as described [28]. Briefly, for nuclei isolation 20-µm-thick sections were deparaffined and underwent subsequent enzyme digestion. Then, 50.000 isolated FFPE nuclei were used in each FFPE-ATAC reaction composed of Tn5-mediated transposition and T7 in vitro transcription. FFPE-ATAC libraries were then sequenced on an Illumina NovaSeq 6000 platform at Novogene (Cambridge, UK) to a depth of at least 40 million 150-bp single-end or paired-end sequencing reads per library.

Statistical analysis

If not reported otherwise, statistical analysis was performed using R (v4.3.0) and p-values were corrected using Benjamini-Hochberg correction. The following R packages were used: TIDYVERSE (v2.0.0)[4] for data handling and plotting; MAFTOOLS (v2.17.0)[29] to summarize, analyze, and visualize variant data; ENHANCEDVOLCANO (v1.18.0) (https://github.com/kevinblighe/EnhancedVolcano) plot volcano plots; COMPLEXHEATMAP (v2.16.0) and PHEATMAP (v1.0.12) to draw heatmaps; GGPUBR (v0.6.0) for box and violin-plots. Intra-tumor heterogeneity was estimated on the entropy of somatic mutation (mDITHER-score) using DITHER (v1.0) [30]. Correlations were calculated using Spearman's rank correlation, if not stated differently.

Pseudonymization

The processing of personal data for this study was performed pseudonymously by using a case ID. Due to pseudonymization data backtracking specific to the individual for non-members of

the study group is nearly impossible. Only the initiators of the study (NG. AK, HW, JS) have access to a file that is separately stored (password-protected) containing the details on pseudonymization.

Declarations

Ethics approval and consent to participate

This retrospective study was approved by the ethics committee of the University of Lübeck (reference-no 18-311) and conducted in accordance with the declaration of Helsinki. Patients at the Reference center for Hematopathology have provided written informed consent regarding routine diagnostic and academic assessment, including genomic studies of their biopsy specimen alongside transfer of their clinical data.

Supplementary Figures – see separate files.

Supplementary Figure 1: Deconvolution of bulk RNA-seq from the extended cohort of BPDCN patients.

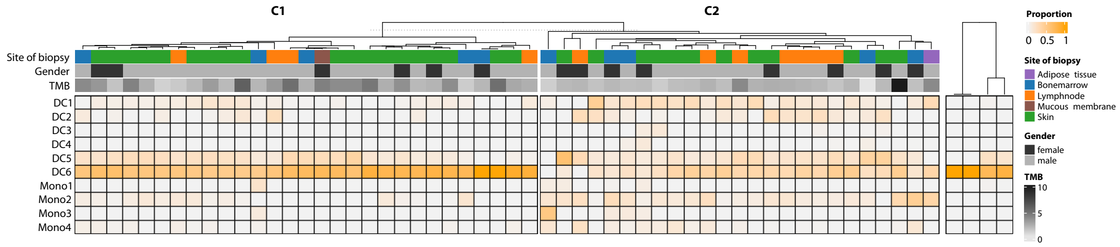

Supplementary Figure 2: Complete representation of promotor and gene body DNA methylation of TSGs (according to Vogelstein *et al.*).

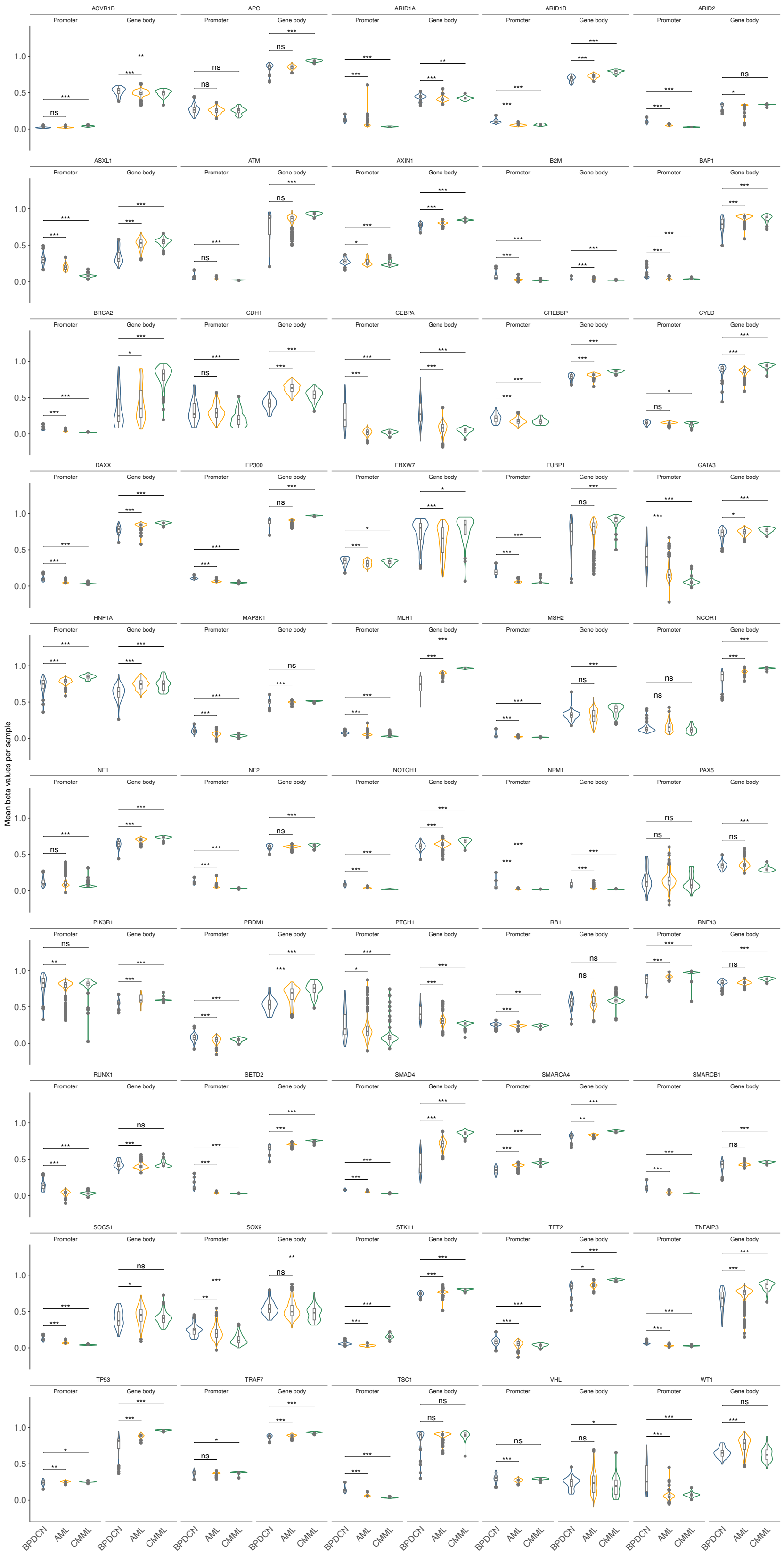

Supplementary Figure 3: Mutational landscape according to BPDCN C1 vs C2. **a** Significantly differentially mutated genes between C1 and C2. **b** TMB according to C1 vs C2

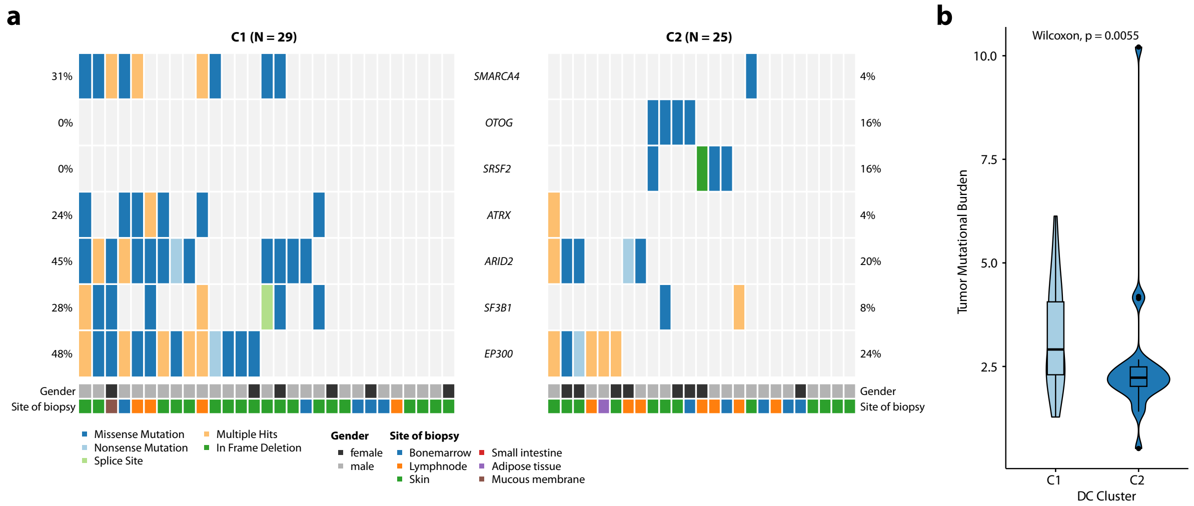

Supplementary Figure 4: Estimated correlation (Pearson's correlation coefficient) between methylated sites and gene expression for gene body and promoter regions of selected genes. Panel **a** displays correlation for genes shown in Figures 3c and 4a and panel **b** shows significant correlations found in tumor suppressor and oncogenes (asterisk denotes significant correlations p < 0.1). Panel **c** shows significant correlations retrieved from a genome-wide scan (asterisk denotes significant correlations p < 0.01 and absolute correlation above 0.1 in either gene body or promoter region); **Supplementary Table 5** lists results from the genome-wide scan.

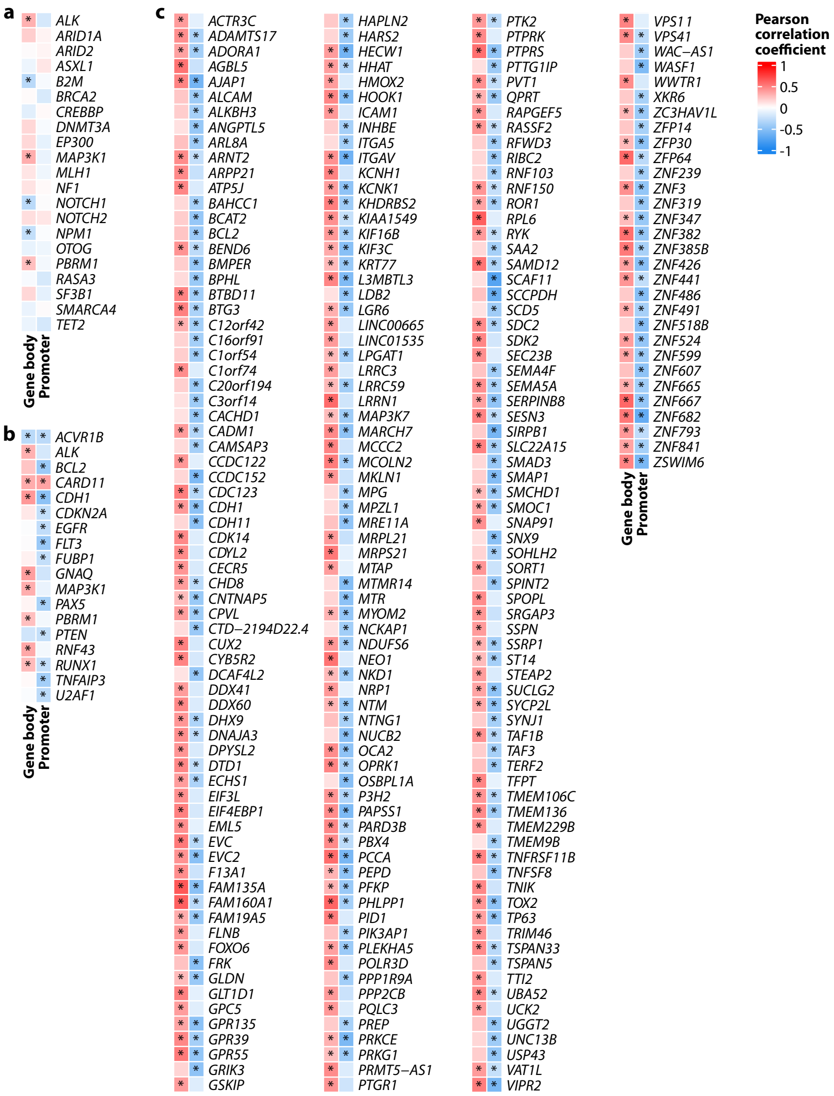

Supplementary Figure 5: Correlating the results from our MethylCIBERSORT approach with the genomic landscape. Mutations in *EP300* were associated with lower levels of monocytes (CD14), effector T-cells (CD4_Eff), and higher levels of regulatory T-cell infiltrates (Treg). While *KMT2C* mutations were associated with fewer eosinophils (Eos) within the TME and *NOTCH2* mutations were found in patients with significantly fewer monocytes (CD14) within their respective TME.

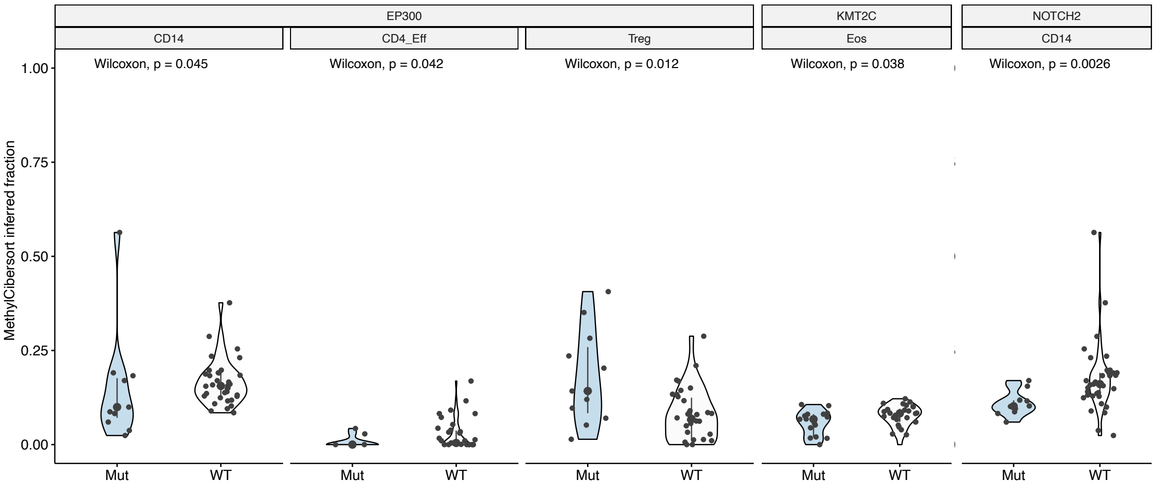

Supplementary Figure 6: Mutational, epigenetic and immunohistochemical properties according to ICs. We observed enrichment in mutations affecting *CDH1*, *ERBB2*, *ASXL1*, *EP300*, *KMT2C, JAK2, SMAD2, NOTCH1*, and *DNMT3A* (**a**) and a trend towards a higher TMB (**b**) in IC1. Similar characteristics in terms of TME composition were observed by immunohistochemistry and MethylCibersort with enrichment of T-cells in IC1 and monocytes in IC2 (**c-f**). epiCMIT was applied to estimate the history of proliferative stress/DNAm age in both immunological clusters (**g**) and revealed significantly higher proliferative stress in IC2 compared to IC1, which resembles DNA methylation-based pre-aging in this subgroup (p = 0.038)

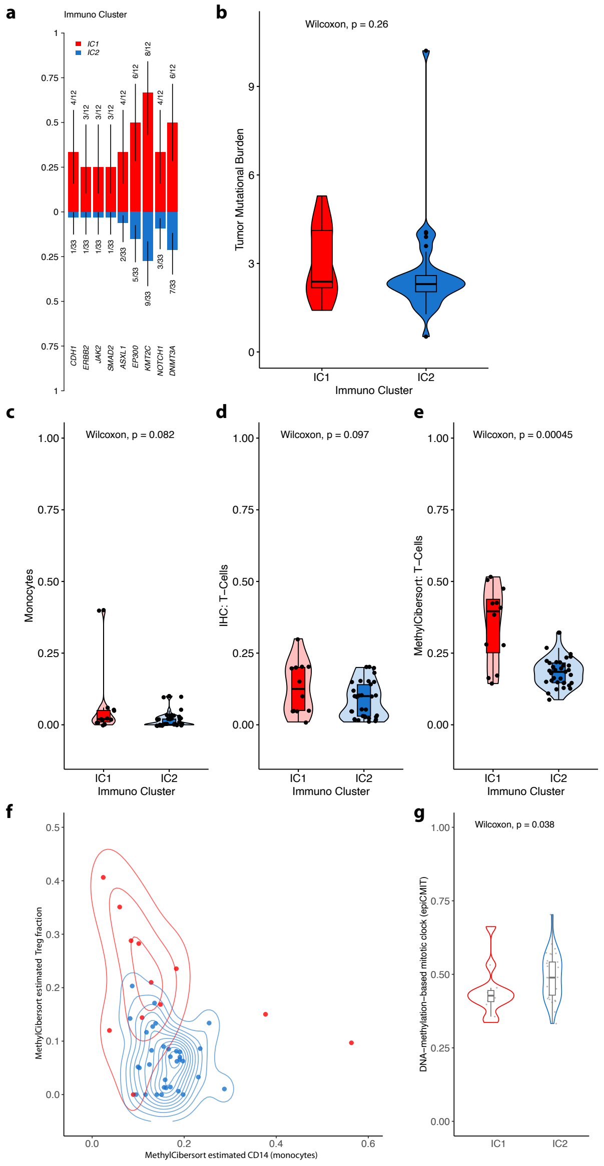

Supplementary Figure 7: Quantitative estimation of tumor-infiltrating T-cells by **a** IHC and **b** MethylCIBERSORT

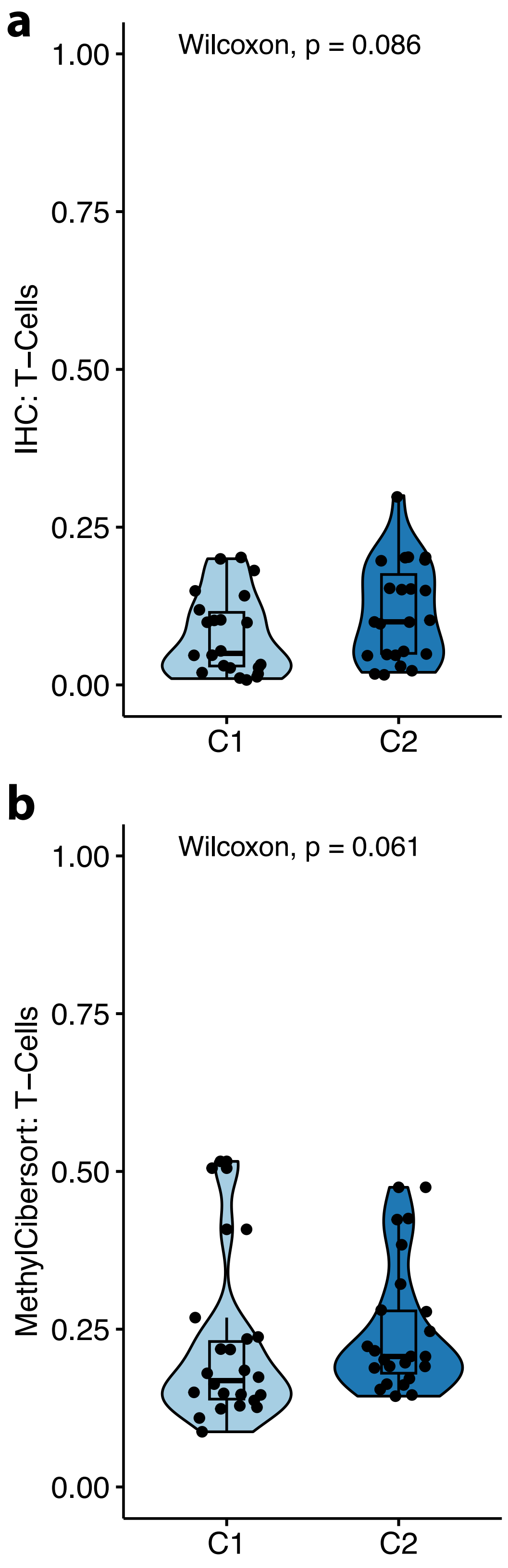

Supplementary Figure 8: Unsupervised clustering of most variable methylated probes (n = 5,600) depicted as **a** heatmap and **b** TMB according to MethCs.

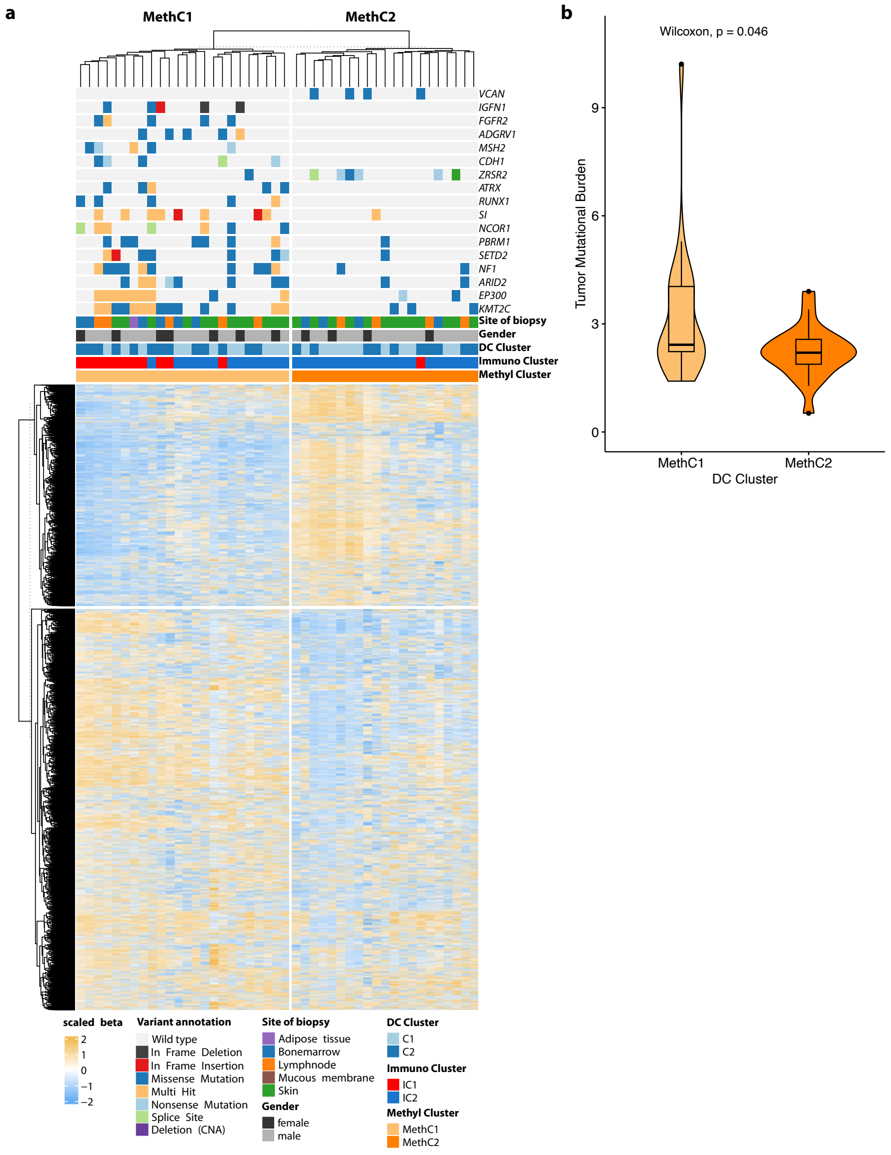

Supplementary Tables – see separate files

Supplementary Table 1: Identified variants after filtering.

Supplementary Table 2: Results retrieved from the MutSigCV analysis.

Supplementary Table 3: Immunohistochemical and molecular data of the study cohort (a), details on *RUNX1*-mutant BPDCN (b), and details on *RUNX1*-mutant AML from the cohort used in comparative analysis (c).

Supplementary Table 4: beta values for differentially methylated probes in the BPDCN cohort.

Supplementary Table 5: Genome-wide scan for correlation between DNA-methylation at gene-body as well as promotor sites and gene expression profiles across the entire transcriptome

Supplementary References

1. Künstner A, Schwarting J, Witte HM, Bernard V, Stölting S, Kusch K*, et al.* Integrative molecular profiling identifies two molecularly and clinically distinct subtypes of blastic plasmacytoid dendritic cell neoplasm. *Blood Cancer Journal* 2022 2022/07/04; **12**(7)**:** 101.

2. Witte HM, Kunstner A, Hertel N, Bernd HW, Bernard V, Stolting S*, et al.* Integrative genomic and transcriptomic analysis in plasmablastic lymphoma identifies disruption of key regulatory pathways. *Blood Adv* 2021 Oct 29.

3. Chen S, Zhou Y, Chen Y, Gu J. fastp: an ultra-fast all-in-one FASTQ preprocessor. *Bioinformatics* 2018 Sep 1; **34**(17)**:** i884-i890.

4. Wickham H, Averick M, Bryan J, Chang W, D'Agostino L, Francois R*, et al.* Welcome to the Tidyverse. *The Journal of Open Source Software* 2019; **4**(43).

5. McKenna A, Hanna M, Banks E, Sivachenko A, Cibulskis K, Kernytsky A*, et al.* The Genome Analysis Toolkit: a MapReduce framework for analyzing next-generation DNA sequencing data. *Genome Res* 2010 Sep; **20**(9)**:** 1297-1303.

6. Sherry ST, Ward MH, Kholodov M, Baker J, Phan L, Smigielski EM*, et al.* dbSNP: the NCBI database of genetic variation. *Nucleic Acids Res* 2001 Jan 1; **29**(1)**:** 308-311.

7. Cibulskis K, Lawrence MS, Carter SL, Sivachenko A, Jaffe D, Sougnez C*, et al.* Sensitive detection of somatic point mutations in impure and heterogeneous cancer samples. *Nat Biotechnol* 2013 Mar; **31**(3)**:** 213-219.

8. McLaren W, Gil L, Hunt SE, Riat HS, Ritchie GR, Thormann A*, et al.* The Ensembl Variant Effect Predictor. *Genome Biol* 2016 Jun 6; **17**(1)**:** 122.

9. Shyr C, Tarailo-Graovac M, Gottlieb M, Lee JJ, van Karnebeek C, Wasserman WW. FLAGS, frequently mutated genes in public exomes. *BMC Med Genomics* 2014 Dec 3; **7:** 64.

10. Vogelstein B, Papadopoulos N, Velculescu VE, Zhou S, Diaz LA, Jr., Kinzler KW. Cancer genome landscapes. *Science* 2013 Mar 29; **339**(6127)**:** 1546-1558.

11. Lawrence MS, Stojanov P, Polak P, Kryukov GV, Cibulskis K, Sivachenko A*, et al.* Mutational heterogeneity in cancer and the search for new cancer-associated genes. *Nature* 2013 Jul 11; **499**(7457)**:** 214-218.

12. Dobin A, Davis CA, Schlesinger F, Drenkow J, Zaleski C, Jha S*, et al.* STAR: ultrafast universal RNA-seq aligner. *Bioinformatics* 2013 Jan 1; **29**(1)**:** 15-21.

13. Yin S, Wang X, Jia G, Xie Y. MIXnorm: normalizing RNA-seq data from formalin-fixed paraffin-embedded samples. *Bioinformatics* 2020 Jun 1; **36**(11)**:** 3401-3408.

14. Ritchie ME, Phipson B, Wu D, Hu Y, Law CW, Shi W*, et al.* limma powers differential expression analyses for RNA-sequencing and microarray studies. *Nucleic Acids Res* 2015 Apr 20; **43**(7)**:** e47.

15. Kaspi A, Ziemann M. mitch: multi-contrast pathway enrichment for multi-omics and single-cell profiling data. *BMC Genomics* 2020 Jun 29; **21**(1)**:** 447.

16. Villani AC, Satija R, Reynolds G, Sarkizova S, Shekhar K, Fletcher J*, et al.* Single-cell RNA-seq reveals new types of human blood dendritic cells, monocytes, and progenitors. *Science* 2017 Apr 21; **356**(6335).

17. Schubert M, Klinger B, Klunemann M, Sieber A, Uhlitz F, Sauer S*, et al.* Perturbation-response genes reveal signaling footprints in cancer gene expression. *Nat Commun* 2018 Jan 2; **9**(1)**:** 20.

18. Pidsley R, Zotenko E, Peters TJ, Lawrence MG, Risbridger GP, Molloy P*, et al.* Critical evaluation of the Illumina MethylationEPIC BeadChip microarray for whole-genome DNA methylation profiling. *Genome Biol* 2016 Oct 7; **17**(1)**:** 208.

19. Roy R, Ramamoorthy S, Shapiro BD, Kaileh M, Hernandez D, Sarantopoulou D*, et al.* DNA methylation signatures reveal that distinct combinations of transcription factors specify human immune cell epigenetic identity. *Immunity* 2021 Nov 9; **54**(11)**:** 2465-2480 e2465.

20. Vento-Tormo R, Company C, Rodriguez-Ubreva J, de la Rica L, Urquiza JM, Javierre BM*, et al.* IL-4 orchestrates STAT6-mediated DNA demethylation leading to dendritic cell differentiation. *Genome Biol* 2016 Jan 13; **17:** 4.

21. Jung N, Dai B, Gentles AJ, Majeti R, Feinberg AP. An LSC epigenetic signature is largely mutation independent and implicates the HOXA cluster in AML pathogenesis. *Nat Commun* 2015 Oct 7; **6:** 8489.

22. Giacopelli B, Wang M, Cleary A, Wu YZ, Schultz AR, Schmutz M*, et al.* DNA methylation epitypes highlight underlying developmental and disease pathways in acute myeloid leukemia. *Genome Res* 2021 May; **31**(5)**:** 747-761.

23. Roels J, Thenoz M, Szarzynska B, Landfors M, De Coninck S, Demoen L*, et al.* Aging of preleukemic thymocytes drives CpG island hypermethylation in T-cell acute lymphoblastic leukemia. *Blood Cancer Discov* 2020 Nov; **1**(3)**:** 274-289.

24. Palomo L, Malinverni R, Cabezon M, Xicoy B, Arnan M, Coll R*, et al.* DNA methylation profile in chronic myelomonocytic leukemia associates with distinct clinical, biological and genetic features. *Epigenetics* 2018; **13**(1)**:** 8-18.

25. Chakravarthy A, Furness A, Joshi K, Ghorani E, Ford K, Ward MJ*, et al.* Pan-cancer deconvolution of tumour composition using DNA methylation. *Nat Commun* 2018 Aug 13; **9**(1)**:** 3220.

26. Duran-Ferrer M, Clot G, Nadeu F, Beekman R, Baumann T, Nordlund J*, et al.* The proliferative history shapes the DNA methylome of B-cell tumors and predicts clinical outcome. *Nat Cancer* 2020 Nov; **1**(11)**:** 1066-1081.

27. Horvath S. DNA methylation age of human tissues and cell types. *Genome Biol* 2013; **14**(10)**:** R115.

28. Zhang H, Polavarapu VK, Xing P, Zhao M, Mathot L, Zhao L*, et al.* Profiling chromatin accessibility in formalin-fixed paraffin-embedded samples. *Genome Res* 2022 Jan; **32**(1)**:** 150-161.

29. Mayakonda A, Lin DC, Assenov Y, Plass C, Koeffler HP. Maftools: efficient and comprehensive analysis of somatic variants in cancer. *Genome Res* 2018 Nov; **28**(11)**:** 1747-1756.

30. Li L, Chen C, Wang X. DITHER: an algorithm for Defining IntraTumor Heterogeneity based on EntRopy. *Brief Bioinform* 2021 Nov 5; **22**(6).
